# Long-term outcomes of modified transcanalicular diode laser dacryocystorhinostomy

**DOI:** 10.5935/0004-2749.2023-0143

**Published:** 2024-03-27

**Authors:** Eduardo Damous Feijó, Juliana Alves Caixeta, Bruna Angelina Alves de Souza, Roberto Murillo Limongi

**Affiliations:** 1 Department of Oculoplastic Surgery, Hospital Oftalmológico de Anápolis, Anápolis, GO, Brazil; 2 Department of Otorhinolaryngology, Universidade Federal de Goiás, Goiânia. GO, Brazil; 3 Department of Oculoplastic Surgery, Universidade Federal de Goiás, Goiânia, GO, Brazil

**Keywords:** Lacrimal duct obstruction, Nasolacrimal duct/surgery, Dacryocystorhinostomy, Lacrimal apparatus diseases, Laser therapy/methods, Lasers, semiconductor/therapeutic use, Regeneration

## Abstract

**Purpose:**

The purpose of this study is to assess the long-term outcomes of modified transcanalicular diode laser dacryocystorhinostomy in a large cohort of patients affected by primary acquired nasolacrimal duct obstruction.

**Methods:**

This study, conducted from January 17 to June 2022, encompassed 141 patients (159 procedures) who underwent modified transcanalicular diode laser dacryocystorhinostomy (MT-DCR). The procedure employed an 810-nm diode laser. Patients were monitored for at least a year after the intervention. Anatomical success was determined by ostium patency upon irrigation, while functional success referred to epiphora resolution. Parameters studied included patient demographics, procedure duration, complications, and both anatomical and functional success. Statistical analysis was performed using the Statistical Package for the Social Sciences software, with results considered significant at a 95% confidence interval (p≤0.05).

**Results:**

A total of 159 lacrimal drainage systems (141 patients: 112 women and 29 men) were included in this study. Among them, 18 underwent bilateral procedures. The average patient age was 58 years (range: 34-91 years), and the average surgical duration was 24 minutes (range: 18-35 minutes). One year after the surgery, MT-DCR exhibited anatomical and functional success rates of 84.9% (135/159) and 83% (132/159), respectively.

**Conclusion:**

MT-DCR achieved an anatomical success rate of 84.9%, reflecting an excellent outcome. However, further extensive studies with larger sample sizes and longer follow-up periods are necessary to substantiate these findings.

## INTRODUCTION

Dacryocystorhinostomy (DCR) stands as the traditional surgical technique for treating primary acquired nasolacrimal duct obstruction (PANDO)^(1^-^4)^. The objective of this procedure is to establish a connection between the lacrimal sac and the nasal mucosa in the middle meatus, allowing permanent drainage through a new drainage pathway^(5^-^7)^. With recent advancements in optic fiber and laser technologies, laser-assisted transcanalicular DCR (T-DCR) has emerged as a minimally invasive alternative^(8^-^11)^. The modified transcanalicular diode laser dacryocystorhinostomy (MT-DCR) involves a blade scalpel removing a rectangular nasal mucosal flap prior to laser osteotomy. This modification aims to mitigate excessive thermal injury, fibrosis, and excessive scarring of the nasal mucosa, which might otherwise result in anatomical or functional complications^(12)^.

This study specifically assesses the long-term outcomes of MT-DCR within a large cohort of patients afflicted with PANDO.

## METHODS

This prospective and interventional study enrolled 141 patients (159 procedures) who underwent MT-DCR from January 2017 to June 2022. Eligible participants met the criteria of experiencing chronic tearing and receiving clinical confirmation of PANDO through methods such as probing, canalicular system irrigation, and dacryocystography. Those excluded were individuals under 18 years old, with secondary lacrimal obstruction, and with history of facial trauma, upper lacrimal obstruction, trichiasis, ectropion, entropion, nasal synechiae, polyps, accentuated nasal septum deviation, and/or middle turbinate hypertrophy.

The procedures were performed by the same surgeons (EDF and JAC), utilizing an 810-nm Fox Laser (Arch™, Germany) coupled with a 23-gauge (G) fiberoptic catheter. This study was carried out at the Oculoplastic Department of the Ophthalmology Hospital of Anápolis, Brazil, spanning from January 2017 to June 2022. The study protocol was approved by the medical research ethics committee, and informed consent was obtained from all participants, in accordance with the International Review Board approval. This study adhered to the guidelines outlined in the Declaration of Helsinki.

### Anesthesia

Every patient was administered ocular topical anesthesia through 1% tetracaine drops, along with nasal anesthesia utilizing a combination of 1% nasal tetracaine drops and a 20% lidocaine spray. Cotton pieces soaked in a 5% naphazoline solution were placed on the nasal mucosa for 5 minutes. Following venous sedation, nerve blocks involving infraorbital and infratrochlear nerves were performed using 2% lidocaine and 0.75%.

### Surgical technique

**Figure 1:**
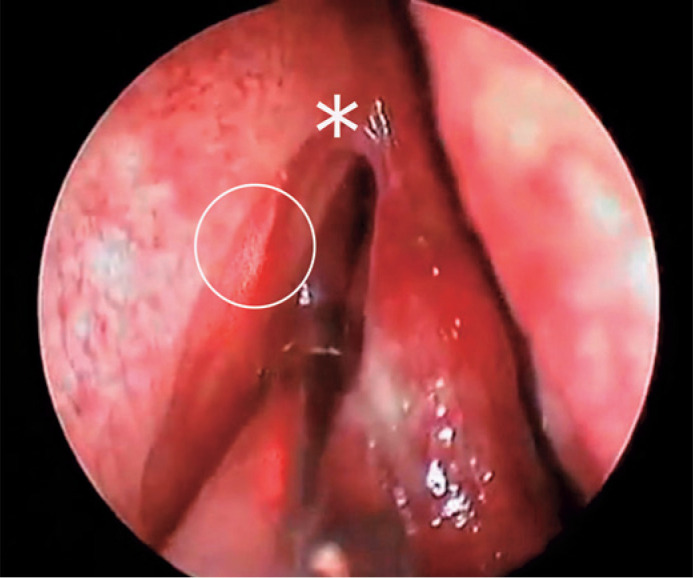
Laser probe reaching the medial wall of the lacrimal sac (asterisk: axilla of the middle turbinate; circle: the laser probe).

**Figure 2:**
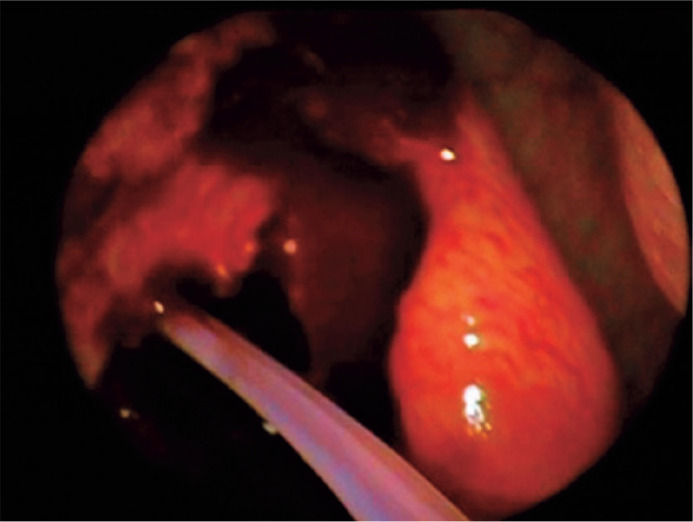
The ostium in the middle meatus with bicanalicular silicone intubation.

The upper and lower lacrimal puncta were dilated using a lacrimal punctum dilator, followed by lacrimal syringing with 0.9% saline solution. Through the superior canaliculus, the 23G endolaser probe connected to the 810-nm diode laser was inserted, reaching the medial wall of the lacrimal sac and the lacrimal bone (Figure 1). A vertical incision was made in the nasal mucosa using a sickle edge blade directly anterior to the maxillary line and extended from the projection of the line of insertion of the middle turbinate to the upper portion of the inferior turbinate. Two relaxing incisions perpendicular to the first incision were made, elevating a nasal mucosal flap using a freer elevator. The flap’s posterior section was incised, and a rectangular portion of the nasal mucosa was removed. Employing the diode laser with parameters of 6W in continuous mode, the osteotomy was performed close to the axilla of the middle tur-binate. The osteotomy size measured approximately 6 millimeters (mm) x 6 mm. Bicanalicular intubation was performed using a silicone tube, which remained in place for 6 weeks (Figure 2). The silicone tube was secured externally beside the nose’s ala through two nylon 6.0 sutures.

Following the procedure, all patients were instructed to use antibiotic drops in combination with corticoste-roids four times daily for a week. Additionally, nasal irrigations with 0.9% saline solution in the operated nostril and nasal corticosteroid (mometasone) spray were applied twice daily for a month. Lacrimal syringing was performed to confirm patency on the 1st, 7th, and 14th postoperative days, coinciding with silicone intubation removal (6 weeks after surgery), at 6 months, and at 1 year after the surgery.

A successful functional outcome of the procedure was defined as either the disappearance or improvement of epiphora (Munk criteria 0.1 or 2) when compared to the preoperative condition. This determination was confirmed through an external eye examination using the dye disappearance test. Anatomical success was established through positive syringing results. The minimum follow-up duration was 1 year (range: 12-36 months). In instances of persistent epiphora, patients underwent nasal endoscopy and dacryocystography to determine potential causes for the procedure’s failure. Statistical analysis was carried out using the Statistical Package for the Social Sciences software (SPSS), with results achieving significance at a 95% confidence interval (p≤0.05).

## RESULTS

A total of 159 lacrimal drainage systems (141 patients: 112 women and 29 men) were included in this study. Among them, 18 underwent bilateral procedures. The average age of the cohort was 58 years (range: 34-91 years), and the average surgical duration was 24 minutes (range: 18-35 minutes) (Table 1). One year after the surgery, MT-DCR exhibited anatomical and functional success rates of 84.9% (135/159) and 83% (132/159), respectively.

**Table 1:** Patient data and anatomical success of the procedure

	MT-DCR (n=159 procedures)
Gender (F/M)	112/29 (141 patients)
Age (Years)	58
Side (R/L)	89/70
Duration (min)	24 min
Surgical sucess	135 (84.9%)
Failure	24 (15.1%)

MT-DCR= modified transcanalicular dacryocystorhinostomy; F= female; M= male; R/L= right/left; min= minutes.

Among the subset of 24 patients with anatomical failure, ophthalmologic and nasal endoscopy examinations revealed cicatricial ostium closure in 22 patients and common canalicular obstruction in 2 patients (Table 2). Despite attaining anatomical success, three patients did not experience improved epiphora. The surgical procedures demonstrated excellent hemostasis, with no occurrences of complications or intranasal structure damage.

**Table 2:** Causes of failure of the procedure

	MT-DCR (n=159 procedures)
Total of failure	24 (15.1% of 159)
Ostium closure	22 (91.6% of 24)
Common canaliculum obstruction	2 (8.4% of 24)

Four cases of silicon tube extrusion before the designated 6-weeks period were identified as postoperative adverse events. However, it is noteworthy that these occurrences did not lead to procedural failures in any of these patients.

## DISCUSSION

There are two main conventional DCR techniques: external DCR, considered the gold standard, and endonasal DCR, which utilizes nasal endoscopy (without facial scarring). Recent studies indicate that the efficacy of these classic techniques ranges from 90% to 95%^(3^,^7^,^13^,^14)^. Classic transcanalicular diode laser DCR (CT-DCR) has displayed varying success rates, spanning from 46% to 90% in previous studies. Typically, the mean success rates are approximately 70-75% within the first year^(9^,^15^-^18)^. One potential explanation for the relatively lower long--term success rates of CT-DCR is attributed to the use of high-power lasers alongside smaller osteotomies. The application of very high energy during rhinostomy procedures results in substantial tissue damage, promoting postoperative fibrosis and occlusion of the ostium due to increased fibroblast activity within the nasal mucosa^(10^-^12^,^18^,^19)^. Numerous attempts have been undertaken to improve surgical success rates, including employing antimetabolites, different laser types, low-energy lasers, and surgical ostium expansion using endonasal forceps^(9^,^15^,^19^-^21)^.

The MT-DCR, proposed by Feijó et al., involves excising nasal mucosa with a blade scalpel instead of a laser, thus preventing mucosal thermal injury, reducing nasal fibrosis, and enhancing results, as indicated by its high efficacy rates^(12^,^22)^.

Limited studies have reported MT-DCR success rates. Two studies comparing CT-DCR to MT-DCR showed success rates of 65-77% and 75-90%, respectively^(12)^. Despite these studies not revealing a statistically significant difference in success rates between CT-DCR and MT-DCR, we believe that the relatively small sample sizes may have contributed to these results. In this case series, we opted for nasal mucosa excision before laser activation and refrained from using mitomycin at the ostium to avoid confounding bias on the results^(12^,^20^,^23)^.

Generally, MT-DCR success rates are lower than those of traditional external and endoscopic DCR. However, due to its minimally invasive nature and short operative time, MT-DCR could be a preferred choice for certain ophthalmologists, especially for young patients without intranasal pathology and coagulation disorders and for elderly patients at risk during general anesthesia. In MT-DCR, effective bleeding control, short operative time, and low laser power are key factors in achieving high success rates^(8^-^12)^.

The authors encountered three patients who did not experience epiphora improvement despite lacrimal patency. This condition, termed functional lacrimal obstruction, can arise due to various factors. In these cases, we believe that all three patients suffered from lacrimal pump malfunction resulting from previous eyelid laxity.

As illustrated in this study, the success rate of MT--DCR was 84.9%, which constitutes an excellent outcome when compared to the established gold standard procedures. However, further comprehensive studies are required, encompassing a larger sample size and a longer follow-up time.
